# High-grade clear cell renal cell carcinoma has a higher angiogenic activity than low-grade renal cell carcinoma based on histomorphological quantification and qRT–PCR mRNA expression profile

**DOI:** 10.1038/sj.bjc.6603796

**Published:** 2007-05-15

**Authors:** M M Baldewijns, V L Thijssen, G G Van den Eynden, S J Van Laere, A M Bluekens, T Roskams, H van Poppel, A P De Bruïne, A W Griffioen, P B Vermeulen

**Affiliations:** 1 Angiogenesis Laboratory, Department of Pathology, Research Institute for Growth and Development (GROW), Maastricht University & University Hospital Maastricht, Maastricht NL 6229 HX, The Netherlands; 2 Translational Cancer Research Group (Lab Pathology University of Antwerp/University Hospital Antwerp, Edegem; Oncology Center, General Hospital St Augustinus, Wilrijk), Antwerp B-2650, Belgium; 3 Department of Pathology, University Hospitals of Leuven, Leuven B-3000, Belgium; 4 Department of Urology, University Hospitals of Leuven, Leuven B-3000, Belgium

**Keywords:** angiogenesis, hypoxia, renal cell carcinoma

## Abstract

Clear cell renal cell carcinoma (CC-RCC) is a highly vascularised tumour and is therefore an attractive disease to study angiogenesis and to test novel angiogenesis inhibitors in early clinical development. Endothelial cell proliferation plays a pivotal role in the process of angiogenesis. The aim of this study was to compare angiogenesis parameters in low nuclear grade (*n*=87) *vs* high nuclear grade CC-RCC (*n*=63). A panel of antibodies was used for immunohistochemistry: CD34/Ki-67, carbonic anhydrase IX, hypoxia-inducible factor-1*α* (HIF-1*α*) and vascular endothelial growth factor (VEGF). Vessel density (MVD – microvessel density), endothelial cell proliferation fraction (ECP%) and tumour cell proliferation fraction (TCP%) were assessed. mRNA expression levels of angiogenesis stimulators and inhibitors were determined by quantitative RT–PCR. High-grade CC-RCC showed a higher ECP% (*P*=0.049), a higher TCP% (*P*=0.009), a higher VEGF protein expression (*P*<0.001), a lower MVD (*P*< 0.001) and a lower HIF-1*α* protein expression (*P*=0.002) than low-grade CC-RCC. Growth factor mRNA expression analyses revealed a higher expression of angiopoietin 2 in low-grade CC-RCC. Microvessel density and ECP% were inversely correlated (Rho=−0.26, *P*=0.001). Because of the imperfect association of nuclear grade and ECP% or MVD, CC-RCC was also grouped based on low/high MVD and ECP%. This analysis revealed a higher expression of vessel maturation and stabilisation factors (placental growth factor, PDGFB1, angiopoietin 1) in CC-RCC with high MVD, a group of CC-RCC highly enriched in low nuclear grade CC-RCC, with low ECP%. Our results suggest heterogeneity in angiogenic activity and vessel maturation of CC-RCC, to a large extent linked to nuclear grade, and, with probable therapeutic implications.

Clear cell renal cell carcinoma (CC-RCC) is the most common carcinoma of the renal tubular epithelium accounting for approximately 75% of cases in surgical series (Kovacs *et al*, 1997). Aberrations of the von Hippel-Lindau (*VHL*) tumour suppressor gene have been shown to be an early and distinct event in the development of CC-RCC (Kaelin, 2004). In up to 70% of CC-RCC, somatic *VHL* gene inactivation occurs (Clifford *et al*, 1998). The *VHL* gene product (pVHL) binds the hypoxia-inducible factor 1*α* (HIF-1*α*) in normoxic cells. Lack of VHL protein leads to stabilisation of HIF-1*α*, a state that is normally seen only in hypoxic cells. The increased levels of HIF-1*α* in CC-RCC (Wiesener *et al*, 2001) are thus mainly caused by genetic alterations of the *VHL* gene in addition to or despite stimulation through hypoxia. Stabilisation of HIF-1*α* activates a cascade of pathways, which include angiogenesis, glycolysis, proliferation and alterations in microenvironmental pH (carbonic anhydrase IX (CAIX)) (Semenza, 1999). Overexpression of angiogenic factors due to HIF-1*α* upregulation can explain the hypervascular nature of CC-RCC.

Tumour angiogenesis is a complex process that is regulated by a balance between pro-angiogenic and anti-angiogenic factors. This balance is influenced by tumour cells and the tumour's microenvironment (Carmeliet and Jain, 2000; Griffioen and Molema, 2000). The main player in angiogenesis is the endothelial cell (EC), and numerous activators of EC proliferation and migration have been described. Most of these activators are receptor kinase ligands, such as vascular endothelial growth factor (VEGF), fibroblast growth factor (FGF), platelet-derived growth factor (PDGF) and epidermal growth factor (EGF), but they can also be of different origin, such as lysophosphatic acid or interleukin-8 (IL8), tumour necrosis factor-*α* (TNF-*α*) (Konig *et al*, 1999; Ruoslahti, 2002; Bergers and Benjamin, 2003). Naturally occurring angiogenic inhibitors include statins (e.g. endostatin, angiostatin), thrombospondin 1 (TSP1) and platelet-derived factor 4 (PF4).

Clear cell renal cell carcinoma has an unpredictable course. The most widely used tumour-related prognostic factors in RCC include stage, Fuhrman nuclear grade and histological type (Gelb, 1997). Additional markers, still under investigation, are cellular proliferation, apoptosis and angiogenesis, among others.

As the highly vascularised phenotype of CC-RCC suggests that angiogenesis is integral to its pathogenesis, the aim of our study was to compare the angiogenic potential of low-grade CC-RCC (Fuhrman grade 1 and 2) with high-grade CC-RCC (Fuhrman grade 3 and 4). To that end, EC proliferation, a measure of ongoing angiogenesis, was immunohistochemically assessed and correlated with vessel density (MVD – microvessel density), tumour cell proliferation, HIF-1*α*, VEGF and CAIX expression. Additionally, the mRNA expression levels of VEGF (-A/B/C), bFGF, placental growth factor (PLGF), angiopoietin 1 (ANG1), angiopoietin 2 (ANG2), PDGFB1, EGF, TNF-*α*, transforming growth factor-*α* (TGF-*α*), transforming growth factor-*β* (TGF-*β*), IL8, PF4, TSP1, vascular endothelial receptors 1 and 2 (VEGFR1 and VEGFR2) were determined within low- and high-grade CC-RCC and correlated with immunohistochemical (IHC) angiogenesis data.

## MATERIALS AND METHODS

### Patients and tissue specimens

Tumour samples of 150 patients with CC-RCC, treated with radical or partial nephrectomy were collected retrospectively. The samples were derived from the archives of the Departments of Histopathology of the University Hospitals of Leuven and Maastricht. None of the patients received any cancer-related therapy before surgery. The original histological slides, stained with haematoxylin and eosin, were reviewed to confirm nuclear Fuhrman grading. All patients were evaluated postoperatively at regular intervals by means of physical examination, chest X-ray, abdominal computed tomography or ultrasound (median follow-up 64 months, range 1–153 months). When indicated, a bone scan was performed. The clinicopathological data of the study population are summarised in Table 1.

### Quantification of MVD, endothelial cell proliferation fraction and tumour cell proliferation fraction

Serial sections of 4 *μ*m were made from the paraffin-embedded kidney tumours. A CD34/Ki67 double-stain procedure was performed on an automated IHC staining system (Dako Autostainer; Dako, Glostrüp, Denmark), as described before (Van den Eynden *et al*, 2005). This technique is used to simultaneously stain ECs (cytoplasmic CD34) and proliferating cells (nuclear Ki-67). Anti-Ki-67 (clone MIB-1, dilution 1/150, Dako, Glöstrup, Denmark) antibody binding was visualised with secondary antibody-labelled polymers containing peroxidase with Diaminobenzidine (DAB) as a substrate. For detection of anti-CD34 (clone QBEnd/10, dilution 1/50, Dako) binding, secondary antibody labelled with an alkaline group and fast red substrate were used. One section was analysed per tumour. For assessment of the vascular density within a tumour section, one hotspot (most vascularised microscopic field) was selected and four areas were chosen randomly. Vessel counts were carried out at × 200 magnification using an optical grid. The presence of a vascular lumen was not necessary to identify a microvessel. Microvessel density was expressed as vessels per mm^2^. Next, a total number of approximately 500 intratumoural ECs and 500 tumour cells were evaluated on consecutive fields at a × 400 magnification and the fractions of proliferating ECs and tumour cells were assessed. Endothelial cell proliferation fraction (ECP%) and tumour cell proliferation (TCP%) were calculated according to the following formulas: ECP%=(the number of ECs with Ki67-stained nuclei/total number of ECs evaluated) × 100; TCP%=(the number of tumour cells with Ki67-stained nuclei/total number of tumour cells evaluated) × 100. Furthermore, in a subgroup of fifteen grade 1 and fifteen grade 4 CC-RCC vessel area was determined by means of Leica Qwin morphometry system (Version 3.2.1, Leica Cambridge, UK).

### Assessment of HIF-1α, CA IX and VEGF

Carbonic anhydrase IX IHC staining was automated using the Dako Autostainer (Dako). Primary antibody anti-CAIX (Rabbit polyclonal, diluted 1/2000, Novus Biologicals, Littleton, CO, USA) was incubated for 60 min at room temperature. Antibody binding was visualised with the ChemMate Envision+detection system (Dako). Semiquantitative analysis of CAIX expression was performed as described before (Chia *et al*, 2001; Chakrabarti *et al*, 2004; Van den Eynden *et al*, 2005). In short, a score of 0–3 for intensity of staining was given (0: no staining, 1: weak staining, 2: moderate staining, 3: strong staining). The percentage of immunostained tumour cells was estimated. The product (intensity score × the percentage of immunoreactive tumour cells) yielded a final score of 0–300.

The HIF-1*α* IHC staining was carried out manually, as described elsewhere (Van den Eynden *et al*, 2005), using an anti-HIF-1*α* primary antibody (Clone 54, diluted 1/500, BD Pharmingen, Franklin Lake, NJ, USA). For HIF-1*α* expression analysis, only cells with completely and darkly stained nuclei were scored as positive. The fraction of HIF-1*α*-positive tumour cells was estimated.

The VEGF IHC staining was performed with Rabbit polyclonal antisera to VEGF reacting with VEGF A isoforms 165 and 121 (Peprotech Inc., Rocky Hill, New York, NY, USA) at a 1/50 dilution. After rinsing with TBS, the slides were blocked for aspecific antibody binding with 1% normal goat serum in TBS for 30 min, followed by incubation with the primary rabbit antihuman VEGF-A antibody. After washing with TBS, the slides were incubated with Powervision poly-HRP goat anti-mouse/rabbit/rat (Immunologic/Klinpath,NL,DPVO-55hrp) for 30 min. Subsequently, an incubation was performed with avidine–biotin complex HRP (Dako) and developed with DAB (Sigma, Zwijndrecht, The Netherlands) for 10 min. The slides were counterstained with haematoxylin (Merck, Darmst, Germany) and mounted for lightmicroscopical evaluation. As negative control, we used slides with normal kidney and RCC tissue, without application of the primary antibody. The staining was semiquantitatively assessed according to a 4-point grading scale: 0 absence of tumour staining, 1+ membrane staining tumour cells, 2+ strong membrane staining and cytoplasmic staining of <50% of the tumour cells and 3+ strong cytoplasmic staining in >50% of all tumour cells.

### RNA isolation, cDNA synthesis and quantitative PCR analysis

From 33 frozen tumour tissues and 16 normal renal tissues, total RNA was isolated from 10 cryosections (20 *μ*m) with the RNeasy mini kit (Qiagen Benelux B.V., Venlo, The Netherlands) according to the supplier's protocol. Possible genomic DNA contamination was removed by on column DNaseI treatment for 20 min at room temperature. Concentration and quality of the RNA was analysed on the NanoDrop ND-1000 (Nanodrop Technologies Inc., Wilmington, USA) and by agarose gel electrophoresis, respectively. A total amount of 100 ng total RNA was used for cDNA synthesis with the iScript cDNA synthesis kit (Bio-Rad Laboratories B.V., Veenendaal, The Netherlands) according to the supplier's protocol. Quantitative PCR was performed with the iCycler (Bio-Rad) in a total volume of 25 *μ*l on 30 ng cDNA with the iQ SYBR Green Supermix (Bio-Rad) and 400 nM forward and reverse primer. The primers used for this study have been described before or were designed and validated as described previously (Thijssen *et al*, 2004). Primers were synthesised by Eurogentec and targeted against cyclophilinA, *β*-actin, 18S rRNA, VEGF (VEGF-A), VEGF-B, VEGF-C, bFGF, PLGF, ANG1, ANG2, EGF, PDGFB1, TNF-*α*, IL8, PF4, TGF-*α*, TGF-*β*, TSP-1, VEGFR1 and VEGFR2.

### Statistical analysis

Statistical analysis was performed using the SPSS 12.0. software package. A *P*-value ⩽0.05 was statistically significant, a 0.05<*P*-value ⩽0.1 was considered a trend toward statistical significance. Normality was tested with a Kolmogorov–Smirnov test assuming normality of data if *P*⩾0.2. If continuous data were normally distributed, correlations were analysed with Pearson's correlation statistics, if not, with Spearman's correlation statistics. In case of normal distribution in all subgroups, equality of means was tested with a Student's *t*-test. If data were not normally distributed, equality of medians was tested with a Mann–Whitney *U*-test. For analysing correlations between categorical variables (e.g. high/low ecp%, high/low mvd, high/low grade), a *χ*^2^ test or, when the assumptions for the *χ*^2^ test were not met, the Fisher's exact test was used.

## RESULTS

### Angiogenesis in low-grade CC-RCC compared with high-grade CC-RCC

**Figure 1:**
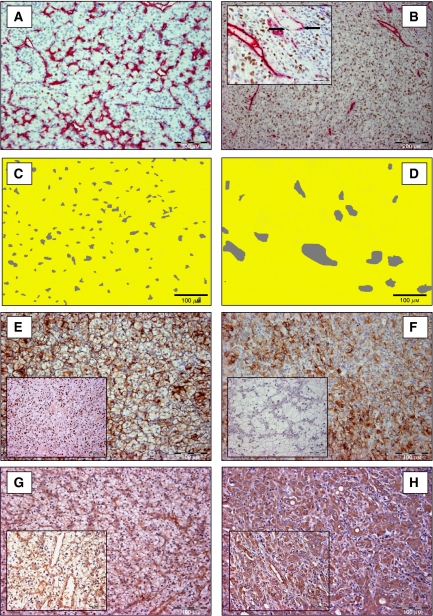
Overview of the IHC stainings used, in low-grade (left: **A, C, E, G**) and high-grade (right: **B, D, F, H**) CC-RCC. (**A** and **B**) CD34/Ki-67 IHC double-staining. Black arrows in inset show proliferating Ki-67 positive (brown nucleus) ECs (red cytoplasm). (**C** and **D**) Morphometrical analyses showing an increased vessel area (grey) in high-grade CC-RCC. (**E** and **F**) Membranous CAIX staining and nuclear HIF-1*α* staining (inset) of tumour cells. Although no significant difference was found between low- and high-grade tumours for CAIX expression, there was more HIF-1*α* expression in low-grade CC-RCC. (**G** and **H**) Predominantly membranous VEGF staining in tumour cells of low-grade RCC (**G**), in contrast with dense cytoplasmic intratumoural VEGF staining in high-grade RCC (**H**). Insets also illustrate VEGF immunoreactivity of the intratumoural vessels.

Median MVD was 181.1 (range 27.6–481.8), median ECP% was 0.7% (range 0–13.2%) and median TCP% was 3.8% (range 0–46.0%). Carbonic anhydrase IX and HIF-1*α* were expressed in 97 and 88% of all tumours, respectively. Vascular endothelial growth factor immunostaining was present in all CC-RCC and was observed mainly in tumour cells and in endothelial cells of intratumoural vessels (Figure 1). Vascular endothelial growth factor immunostaining was graded 1+ in 59 tumours (39.3%), 2+ in 66 tumours (44%) and 3+ in 25 tumours (16.7%). Vascular endothelial growth factor protein expression was inversely related to MVD (Rho=−0.42, *P*<0.001). There was an inverse correlation between MVD and Fuhrman grade (Rho=−0.40, *P*<0.001). Endothelial cell proliferation fraction had a moderate positive correlation with Fuhrman grade (Rho=0.17, *P*=0.029). Microvessel density and ECP% were inversely correlated (Rho=−0.26, *P*=0.001).

**Figure 2:**
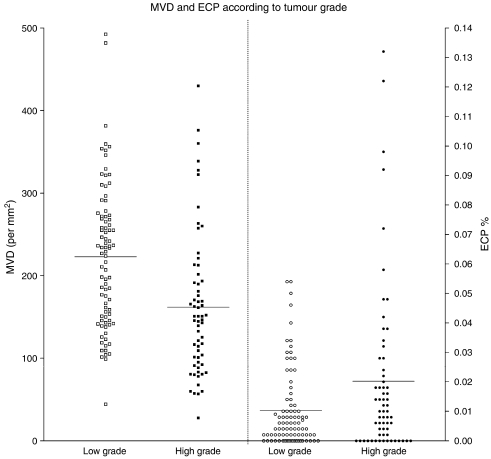
Microvessel density and ECP% according to tumour grade. ECP%: endothelial cell proliferation fraction (%). MVD: microvessel density (vessels per mm^2^).

Based on these results, the group of CC-RCC was split into two groups: high grade (Fuhrman grade 3 and 4) and low grade (Fuhrman grade 1 and 2). Table 2 shows the differences in angiogenesis parameters and hypoxia-related parameters between both sub-populations. High-grade CC-RCC had higher ECP% (Figure 2), higher TCP%, larger vessel area and higher VEGF protein expression. In contrast, MVD, CAIX protein expression and HIF-1*α* protein expression were lower in high-grade tumours.

Tables 3 and 4 illustrate the expression of angiogenic growth factors/inhibitors in CC-RCC and normal renal tissue. In renal tumour tissues, there was a significant overexpression of VEGF (*P*<0.001), VEGF-B (*P*=0.004), VEGF-C (*P*=0.032), PLGF (*P*<0.001), ANG2 (*P*<0.001), PDGFB1 (*P*=0.009) and TGF-*α* (*P*<0.001) compared to normal renal tissue (Table 3). In contrast, renal cell carcinoma expressed less bFGF (*P*<0.001) and EGF (*P*<0.001) than normal renal tissue.

When comparing the PCR data of the angiogenic growth factors in low- and high-grade tumours (Table 4), only a significant difference could be found in ANG2 expression, which was higher in low-grade tumours (*P*=0.035). Low-grade tumours also expressed more VEGFR1 and 2 than high-grade CC-RCC (*P*=0.023 and *P*=0.004, respectively).

### mRNA expression of angiogenesis-related factors in CC-RCC grouped according to median MVD and median ECP%

**Figure 3:**
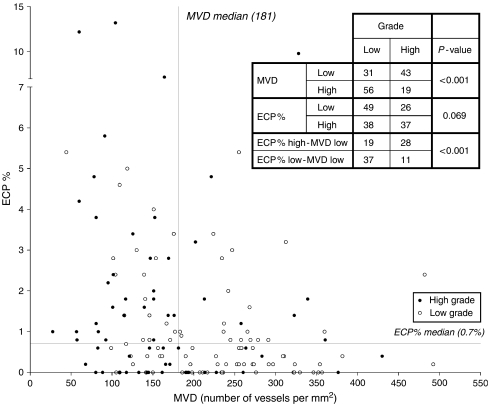
Scatter plot of low-grade (open dots) and high-grade (filled dots) CC-RCC according to median MVD and median ECP%. Tumours with low ECP% and high MVD are mainly low-grade CC-RCC. Tumours with high ECP% and low MVD are mainly high-grade CC-RCC.

Because of the imperfect association of nuclear grade and ECP% or MVD (Figure 3) and to get more insight in the biology of both vascular patterns related to the inverse association of MVD and ECP%, CC-RCC were also grouped based on low/high MVD (cutoff median MVD of 181) and high/low ECP% (cutoff median ECP% of 0.7%). In the CC-RCC with high MVD, there was a higher mRNA expression of VEGF (*P*=0.007), ANG2 (*P*<0.001), PLGF (*P*=0.007) and PDGFB1 (*P*=0.001). There was also more mRNA expression of VEGFR1 and VEGFR2 in the high MVD group (*P*=0.001 and *P*<0.001). A higher expression of ANG1 was found in tumours with low ECP% (*P*=0.008).

## DISCUSSION

The interest of our study was to compare angiogenesis parameters in low-grade *vs* high-grade CC-RCC. In earlier studies, MVD was used as a parameter for angiogenesis in RCC and evaluated as a possible prognostic marker, however leading to conflicting results (Gelb *et al*, 1997; Nativ *et al*, 1998; Rioux-Leclercq *et al*, 2001; Sabo *et al*, 2001). To our knowledge, this is the first study in which the fraction of proliferating ECs (ECP%) is investigated within RCC.

High-grade CC-RCCs were characterised by a high ECP%, which correlated strongly with TCP%. In spite of the higher ECP% in high-grade CC-RCC than the low-grade subgroup, MVD was lower in high-grade CC-RCC. These results suggest that ECP% is a better indicator for ongoing angiogenesis in CC-RCC than MVD. Vessel density reflects intercapillary distance, which is determined by angiogenic factors (stimulators and inhibitors), as well as nonangiogenic factors, such as oxygen and nutrient consumption rates of tumour cells (Hlatky *et al*, 2002). Tumour progression is associated with a gradual reduction in the intrinsic propensity of cancer cells to undergo apoptosis under a variety of noxious conditions (Bedi *et al*, 1995). Cumulative effects of genetic alterations, by means of altered expression of tumour suppressor genes (*PTEN, p53* and *VHL*) and oncogenes (ras,src) could affect HIF-1-dependent and -independent pathways of cellular response to hypoxia (Semenza, 2000). In patients with localised RCC, p53 appeared to be an independent predictor of tumour progression. (Shvarts *et al*, 2005). As a relative decrease of vascular dependence of cancer cells is associated with features of increased malignancy (Carmeliet *et al*, 1998; Yu *et al*, 2001), tolerance of hypoxic conditions can explain why high-grade renal tumours can afford an increased intercapillary distance in comparison with low-grade RCC.

In our study, 88% of all CC-RCC showed activation of HIF1-*α* throughout the whole tumour section, independently of the presence of necrosis. This is consistent with the fact that in 70% of CC-RCC there is a constitutive activation of HIF, through VHL inactivation (Clifford *et al*, 1998). Nevertheless differences in HIF-1*α* expression were seen between low- and high-grade CC-RCC. The lower HIF-1*α* expression in high-grade CC-RCC could possibly be explained by a progressive switch to HIF-2*α* response during tumour progression. An earlier report stated that normal renal epithelium *in vivo* activates HIF-1*α* in response to hypoxia and VHL loss with little or no HIF-2*α* (Rosenberger *et al*, 2002). Hypoxia-inducible factor-2*α* appears to become progressively more evident than HIF-1*α* in foci of renal dysplasia, cyst formation and frank tumours (Mandriota *et al*, 2002). As HIF-1*α* and HIF-2*α* have contrasting properties (Raval *et al*, 2005), a possible switch to HIF-2*α* could increase the likelihood to proliferation and survival, during tumour evolution.

Evaluation of angiogenesis growth factor/inhibitor expression levels between low- and high-grade CC-RCCs, revealed only a significant difference in ANG2 expression, which was significantly higher in the low-grade CC-RCC group. Angiopoietin 2 expression is often induced in endothelia undergoing active remodelling or regression and is upregulated by hypoxia and several growth factors, including VEGF (Oh *et al*, 1999; Mandriota *et al*, 2000). Administration of inhibitors of ANG2 to tumour-bearing mice has been reported to result in delayed tumour growth and reduced EC proliferation. Therefore, inhibitors of ANG2 may be candidates for clinical development (Oliner *et al*, 2004).

In our study, there is a discrepancy between the IHC VEGF data and mRNA VEGF expression. Low-grade CC-RCC with high MVD are characterised by a lower VEGF protein expression and higher mRNA VEGF expression than high-grade CC-RCC with low MVD. This can be explained by the fact that in the IHC VEGF staining we only scored cytoplasmic VEGF positivity in tumour cells, whereas mRNA VEGF expression also includes VEGF in stroma and at the surface of ECs. In our case, CC-RCC with high MVD showed VEGF positive staining of the numerous tumoural vessels (Figure 1).

Within our group of 150 CC-RCCs, the association of MVD and ECP% with nuclear grade was however imperfect. Therefore, to get more insight in angiogenesis biology, we compared the mRNA expression levels of angiogenesis-related factors between two subgroups, based on MVD and ECP%, respectively, and leaving Fuhrman grade out of account. Clear cell renal cell carcinoma with high MVD revealed a significant higher PLGF and PDGFB1 expression. Placental growth factor, a VEGF-related factor, contributes to tumour angiogenesis by providing increased survival function to ECs (Adini *et al*, 2002). PDGFB is required for recruitment of pericytes and maturation of microvasculature (Lindahl *et al*, 1997). Furthermore, tumours with low ECP%, revealed a higher ANG1 expression, which competes with ANG2 for binding of Tie-2 receptor. Angiopoietin 1 promotes EC sprouting and is essential for maturation and stabilisation of the developing vascularisation (Papapetropoulos *et al*, 1999; Distler *et al*, 2003). These findings suggest that CC-RCC with high MVD and low ECP% may have a more stabilised and mature vasculature than their counterpart. Our finding is concordant with a recent study, which described differentiated and undifferentiated types of blood vessels in CC-RCC (Yao *et al*, 2007). They found that an increased density of undifferentiated vessels significantly correlated with higher pathological grades and shorter patient survival.

As immature vasculature has been shown to be more vulnerable for therapeutic targeting (Feron, 2004), a better response to anti-angiogenic drugs might be expected in the high-grade CC-RCC. Selection of resistant tumour cells can however still occur (Yu *et al*, 2002). On the other hand, anti-VEGF treatment probably has direct effects on tumour cells of CC-RCC because these cells express KDR that is phosphorylated, independent of grade (Fox *et al*, 2004).

In summary, the results of this study suggest that high-grade CC-RCC have a different angiogenic pattern than low-grade CC-RCC, with more EC proliferation in the former tumours, but lower MVD, with larger and more immature vessels. These differences in angiogenesis biology might have impact on the effects of anti-angiogenic or anti-VEGF treatment of CC-RCC.

## Figures and Tables

**Table 1:** Clinical and histopathological characteristics

	**Clinicopathological data (*N*=150)**
Gender male/female	99/51 (66/34%)
Age (years)	66.6 (±12.1 s.d.)
Mean tumour diameter (cm)	6.36 (±3.45 s.d.)
Median tumour diameter (cm)	6.00 (0.7–20.0 range)
Fuhrman grade	
1	15 (10.0%)
2	72 (48.0%)
3	46 (30.7%)
4	17 (11.3%)
Tumour status^a^	
T1	82 (54.7%)
T2	24 (16.0%)
T3	42 (28.0)
T4	2 (1.3%)
Lymph node status^a^	
1	4
2	11

a T and N status were assigned according to the TNM classification of the International Union Against Cancer 2002.

**Table 2:** Immunohistochemical and morphometric results of angiogenesis parameters in low-grade *vs* high-grade CC-RCC

		**MVD^a^**	**Hotspot**	**ECP%**	**TCP%**	**CAIX**	**HIF**	**Vessel area^b^**	**VEGF^c^**
Low-grade RCC	Min	44.220	52.030	0%	0%	0	0%	54.99	1: 57.5%
	Median	233.870	297.865	0.6%	2.5%	285	40%	122.80	2: 39.1%
	Max	481.790	741.420	5.4%	30.9%	300	100%	229.83	3: 3.4%
High-grade RCC	Min	27.580	49.430	0%	0%	0	0%	79.66	1: 14.3%
	Median	150.880	250.520	1%	5%	240	15%	148.44	2: 50.8%
	Max	429.760	590.530	13.2%	46%	300	95%	588.30	3: 34.9%
*P*-value		<0.001	<0.001	0.049	0.009	0.262	0.002	0.017	<0.001

Abbreviations: CAIX=carbonic anhydrase IX; CC-RCC=clear cell renal cell carcinoma; ECP%=endothelial cell proliferation fraction; HIF=hypoxia-inducible factor; MVD=microvessel density; RCC=renal cell carcinoma; TCP%=tumour cell proliferation fraction; VEGF=vascular endothelial growth factor.

*P*-value: comparison between low-grade and high-grade RCC (Mann–Whitney).

a MVD expressed as vessels per mm^2^.

b Vessel area expressed as *μ*m^2^.

c For VEGF, the percentage of each of the scores is represented.

**Table 3:** Results of PCR quantification of angiogenesis-related genes in normal renal tissue versus CC-RCC

	**VEGFA**	**VEGFB**	**VEGFC**	**BFGF**	**PLGF**	**ANG1**	**ANG2**	**EGF**	**PDGFB1**	**TNF*α***
Normal										
N	16	16	16	16	16	16	16	16	16	16
Min	0.055	0.012	0.003	0.092	0.002	0.007	0.001	0.004	0.009	0.000
Median	**0.124**	**0.020**	**0.004**	**0.882**	**0.006**	**0.010**	**0.003**	**0.048**	**0.018**	**0.001**
Max	0.334	0.026	0.014	1.717	0.011	0.053	0.0138	0.268	0.057	0.011
										
RCC										
N	33	33	33	33	33	33	33	33	33	33
Min	0.091	0.008	0.001	0.002	0.007	0.000	0.006	0.000	0.008	0.000
Median	**1.187**	**0.035**	**0.008**	**0.045**	**0.114**	**0.007**	**0.045**	**0.001**	**0.037**	**0.002**
Max	4.326	0.209	0.073	0.251	1.945	0.098	0.123	0.042	0.220	0.006
*P*-value	<0.001	0.004	0.032	<0.001	<0.001	0.486	<0.001	<0.001	0.009	0.703
										
	**IL8**	**TGF*α***	**TGF*β***	**TSP1**	**PF4**	**VEGFR1**	**VEGFR2**			
Normal										
N	16	16	16	16	16	16	16			
Min	0.001	0.004	0.023	0.003	0.000	0.001	0.007			
Median	**0.003**	**0.009**	**0.241**	**0.158**	**0.000**	**0.008**	**0.016**			
Max	0.037	0.015	1.515	0.489	0.000	0.018	0.035			
										
RCC										
N	33	33	33	33	33	33	33			
Min	0.000	0.000	0.041	0.007	0.000	0.000	0.002			
Median	**0.007**	**0.038**	**0.260**	**0.227**	**0.000**	**0.012**	**0.030**			
Max	0.110	0.169	1.148	2.158	0.000	0.052	0.205			
										
*P* value	0.875	<0.001	0.745	0.686	0.884	0.035	0.121			

Abbreviations: ANG1=angiopoietin 1; ANG2=angiopoietin 2; BFGF=basic fibroblast growth factor; CC-RCC=clear cell renal cell carcinoma; EGF=epidermal growth factor; PDGFB1=platelet-derived growth factor-B1; PF4=platelet-derived factor 4; PLGF; placental growth factor; RCC=renal cell carcinoma; TNF=tumour necrosis factor; TSP1=thrombospondin 1; VEGFA=vascular endothelial growth factor-A; VEGFB=vascular endothelial growth factor-B; VEGFC=vascular endothelial growth factor-C.

Expression values are shown as 2^−Δ*C*t^.

*P*-value: comparison between normal and RCC (Mann–Whitney).

**Table 4:** Results of PCR quantification of angiogenesis-related genes in low-grade *vs* high-grade CC-RCC

	**VEGFA**	**VEGFB**	**VEGFC**	**bFGF**	**PLGF**	**ANG1**	**ANG2**	**EGF**	**PDGFB1**	**TNF*α***
Low-grade RCC										
N	23	23	23	23	23	23	23	23	23	23
Min	0.094	0.008	0.001	0.002	0.009	0.001	0.012	0.000	0.009	0.000
Median	**1.187**	**0.044**	**0.008**	**0.045**	**0.130**	**0.009**	**0.053**	**0.001**	**0.048**	**0.002**
Max	4.326	0.209	0.073	0.250	1.945	0.098	0.123	0.034	0.220	0.006
										
High-grade RCC										
N	10	10	10	10	10	10	10	10	10	10
Min	0.091	0.008	0.003	0.009	0.007	0.000	0.006	0.000	0.008	0.000
Median	**0.953**	**0.028**	**0.008**	**0.034**	**0.030**	**0.004**	**0.027**	**0.000**	**0.017**	**0.002**
Max	4.060	0.119	0.049	0.251	0.360	0.028	0.063	0.042	0.167	0.005
										
*P*-value	0.597	0.208	0.968	0.393	0.113	0.464	0.035	0.122	0.167	0.903
										
	**IL8**	**TGF*α***	**TGF*β***	**TSP1**	**PF4**	**VEGFR1**	**VEGFR2**			
Low-grade RCC										
N	23	23	23	23	23	23	23			
Min	0.000	0.000	0.041	0.007	0.000	0.001	0.006			
Median	**0.007**	**0.038**	**0.239**	**0.181**	**0.000**	**0.019**	**0.041**			
Max	0.045	0.169	1.148	0.757	0.000	0.052	0.205			
										
High-grade RCC										
N	10	10	10	10	10	10	10			
Min	0.000	0.000	0.043	0.030	0.000	0.000	0.002			
Median	**0.006**	**0.038**	**0.391**	**0.258**	**0.000**	**0.007**	**0.010**			
Max	0.110	0.113	0.972	2.158	0.000	0.034	0.042			
										
*P*-value	0.839	0.871	0.371	0.776	0.371	0.023	0.004			

Abbreviations: ANG1=angiopoietin 1; ANG2=angiopoietin 2; bFGF=basic fibroblast growth factor; CC-RCC=clear cell renal cell carcinoma; EGF=epidermal growth factor; IL8=interleukin-8; PDGFB1=platelet-derived growth factor-B1; PF4=platelet-derived factor 4; PLGF; placental growth factor; RCC=renal cell carcinoma; TGF-*α*=transforming growth factor-*α*; TNF-*α*=tumour necrosis factor-*α*; TSP1=thrombospondin 1; VEGFA=vascular endothelial growth factor-A; VEGFB=vascular endothelial growth factor-B; VEGFC=vascular endothelial growth factor-C.

Expression values are shown as 2^−Δ^.

*P*-value: comparison between low-grade and high-grade RCC (Mann–Whitney).
